# Single metastasis of myxoid liposarcoma from the thigh to thyroid gland: a case report

**DOI:** 10.1186/s12957-018-1370-1

**Published:** 2018-03-27

**Authors:** Hiroshi Urakawa, Kenichi Nakanishi, Eisuke Arai, Kunihiro Ikuta, Shunsuke Hamada, Takehiro Ota, Naoki Ishiguro, Yoshihiro Nishida

**Affiliations:** 1 Department of Orthopaedic Surgery, Nagoya University Graduate School and School of Medicine, 65 Tsurumai, Showa-ku, Nagoya, Aichi 466-8550 Japan; 2 Department of Breast and Endocrine Surgery, Nagoya University Graduate School and School of Medicine, Nagoya, Japan

**Keywords:** Myxoid liposarcoma, Metastasis, Thyroid gland

## Abstract

**Background:**

Thyroid metastasis of soft tissue sarcoma is very rare, and the diagnosis is especially difficult when only a single lesion is present.

**Case presentation:**

A 50-year-old man was diagnosed with myxoid liposarcoma of the right thigh and treated with wide resection. Two and a half years after the surgery, a growing low-density area was incidentally observed in the right lobe of his thyroid gland on follow-up chest computed tomography. Fine needle aspiration biopsy was performed twice, and the thyroid mass was suspected of being a sarcoma metastasis. He was treated by hemithyroidectomy, and the lesion was pathologically confirmed as a metastasis of myxoid liposarcoma.

**Conclusion:**

We experienced single thyroid gland metastasis in patients with myxoid liposarcoma in whom a growing mass is observed in the thyroid gland after radical surgery of the primary site.

## Background

Metastasis to the thyroid gland has been reported in 0.4–1.5% of patients undergoing surgery for malignant thyroid tumors [1, 2], but thyroid gland metastasis is very rare in myxoid liposarcoma, and its clinical features are not well recognized.

Soft tissue sarcoma is a highly metastatic tumor, whose most common metastatic site is the lung, but myxoid liposarcoma is recognized to metastasize to unusual locations such as soft tissue, retroperitoneum, and spine [3]. The reason for metastasis to these peculiar lesions has not been elucidated. A previous report suggested that myxoid liposarcoma has a high incidence of osseous metastases and often associated with the most common type of type II *TLS-CHOP* transcript [4]. Myxoid liposarcoma is known as a translocation-related sarcoma, and there is a possibility that the specific fusion gene may affect the metastatic organotropism. We incidentally detected a single thyroid gland metastasis of myxoid liposarcoma on follow-up chest computed tomography (CT) after definitive surgery and report here a rare myxoid liposarcoma thyroid metastasis from a thigh in an adult.

## Case presentation

**Fig. 1:**
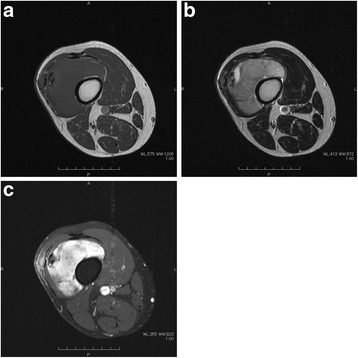
Magnetic resonance T1-weighted image (**a**), T2-weighted image (**b**), and enhanced image with fat suppression (**c**) of primary myxoid liposarcoma of right thigh after open biopsy

**Fig. 2:**
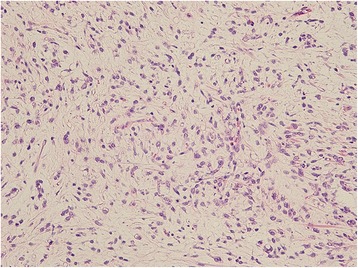
Microphotograph of biopsy specimen in resected primary site of right thigh (H&E, × 200). A mixture of uniform oval shaped non-lipogenic cells and small signet ring lipoblasts in a prominent myxoid stroma were observed

**Fig. 3:**
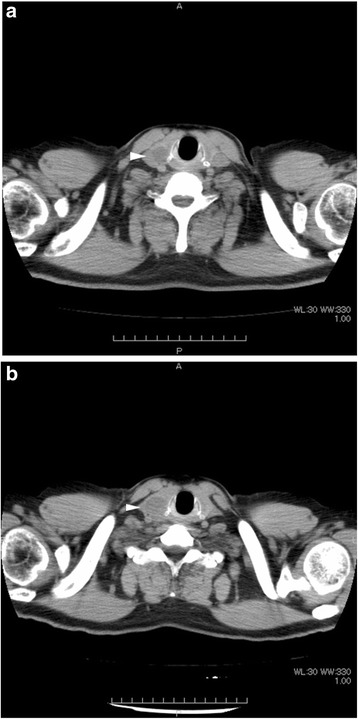
Computed tomography after two and half years (**a**) and three and half years (**b**) after definitive surgery for primary tumor. The white arrowhead indicates a growing low-density area in the right lobe of the thyroid gland

**Fig. 4:**
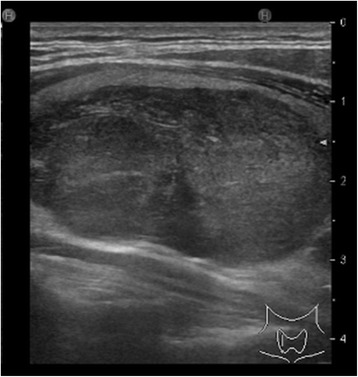
Ultrasound showed a solid hypoechoic nodule with taller than wide shape in the right robe of the thyroid gland

**Fig. 5:**
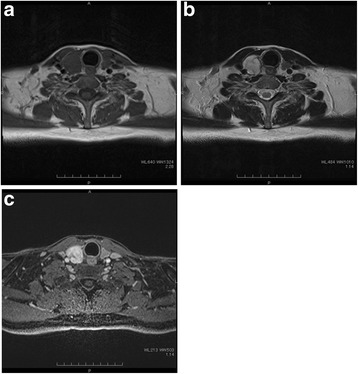
Magnetic resonance T1-weighted image (**a**), T2-weighted image (**b**), and enhanced image with fat suppression (**c**) of metastatic thyroid tumor

**Fig. 6:**
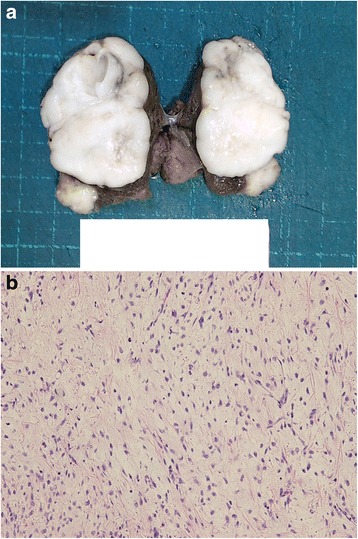
Gross appearance (**a**) and microphotography (H&E, × 200) (**b**) of resected specimen in thyroid gland metastasis after hemithyroidectomy

A 50-year-old man consulted an orthopedic clinic with a chief complaint of a painless mass in his right thigh. He consulted our university hospital 2 weeks later. He was suspected of having a localized myxoid liposarcoma of the right thigh by clinical imaging (Fig. 1a–c) and open biopsy. The maximum tumor diameter was 10 cm. Myxoid liposarcoma was suspected in histological examinations of the biopsy specimens. He was treated by wide resection which involved tumor, vastus intermedius, and vastus lateralis. Histological examination of the resected specimens revealed a mixture of uniform oval shaped non-lipogenic cells and small signet ring lipoblasts in a prominent myxoid stroma, and he was diagnosed with myxoid liposarcoma (Fig. 2). There was no round cell component, and the surgical margin was negative. Two and half years after the surgery, a growing low-density area was incidentally observed in the right lobe of his thyroid gland on follow-up chest CT (Fig. 3a, b). Ultrasound showed a solid hypoechoic nodule with taller than wide shape in the right robe of his thyroid gland (Fig. 4). Ultrasound-guided fine needle aspiration biopsy was performed twice (first biopsy: undeterminable and second biopsy: false-positive), and the thyroid mass was suspected of being a sarcoma metastasis. Magnetic resonance imaging (MRI) showed that the mass in the right lobe of his thyroid gland was isointense on T1-weighted images, hyperintense on T2-weighted images, and well enhanced (Fig. 5a–c). He was treated by hemithyroidectomy (Fig. 6a, b), and the lesion was pathologically confirmed as a metastasis of myxoid liposarcoma. Round component was also not observed in the metastatic myxoid liposarcoma. Postoperative chemotherapy was not done because patient consent was not obtained. There has been no recurrence at one and a half years after the hemithyroidectomy.

## Discussion and conclusions

Recent reports of non-small round cell soft tissue sarcoma metastasis from an extremity to the thyroid gland are listed in Table 1. Six cases of non-small round cell soft tissue sarcoma metastasis from an extremity to the thyroid gland were reported in this century [2, 5–9]. Of these six cases, four had liposarcoma and one of them was diagnosed with myxoid liposarcoma like our case. Especially, a thyroid mass after resection of liposarcoma should be considered a possible thyroid metastasis. Five of seven sarcoma metastases to the thyroid gland occurred in the right lobe in Table 1, but the reason is unknown because the thyroid gland has no left-right difference in weight and blood supply [10].

**Table 1:** Recent reports of non-small round cell soft tissue sarcoma metastasis from extremity to the thyroid gland

Authors	Year	Age^a^	Sex	Primary site	Histological diagnosis	Laterality of the thyroid lobe	Note
Bashir et al. [4]	2002	58	Female	Buttock	Pleomorphic liposarcoma	Right	
Azar et al. [5]	2003	30	Female	Thigh	Pleomorphic liposarcoma	Right	
Brandwein-Gensler et al. [6]	2004	86	Female	Thigh	Liposarcoma	Left	Collison tumor
Wood et al. [2]	2004	71	Unknown	Thigh	Leiomyosarcoma	Unknown	
Tysome et al. [7]	2006	51	Male	Femor	Myxoid liposarcoma	Right	
Vankalakunti et al. [8]	2008	30	Male	Ankle	UPS/MFH	Right	
Our case		52	Male	Thigh	Myxoid liposarcoma	Right	

*UPS* undifferentiated pleomorphic sarcoma, *MFH* malignant fibrous histiocytoma

^a^Age at diagnosis of thyroid gland metastasis

Myxoid liposarcoma without round cell component is considered to be a generally low-grade tumor, with 5-year disease-specific and disease-free survivals of 90 and 77% in our sarcoma group, respectively [11]. Histological diagnosis showed no round cell component in the primary myxoid liposarcoma of the thigh, but thyroid gland metastasis occurred at two and half years after definitive surgery. Interestingly, there was no round cell component in the resected metastatic myxoid liposarcoma of thyroid gland. Wood et al. reported 15 cases of thyroid metastasis, 9 of which had other metastases as well [2]. In our case, the presence of only a single thyroid metastasis made it difficult to diagnosis.

On ultrasound imaging for thyroid mass, the presence of a solid hypoechoic nodule or solid hypoechoic component of a partially cystic nodule with one or more of the following features such as irregular margins, microcalcifications, taller than wide shape, rim calcifications with small extrusive, soft tissue component, and evidence of extra thyroidal extension were recognized as indicating a high risk of malignancy [12]. In this case, ultrasound showed a solid hypoechoic nodule with taller than wide shape in the right robe of his thyroid gland, suggesting the possibility of malignant thyroid tumor. However, it was difficult to distinguish primary thyroid tumor from thyroid metastasis on images.

In our case, there were two other differential diagnoses in thyroid metastasis on MRI, namely primary thyroid tumors such as follicular adenoma and adenocarcinoma. Most of these tumors show similar MRI features to those of myxoid liposarcoma such as isointensity on T1-weighted images and hyperintensity on T2-weighted images [13], making it difficult to distinguish a sarcoma metastasis from these follicular tumors without biopsy. Fine needle aspiration biopsy is known to be the most accurate and cost-effective method for evaluating thyroid nodules [12]. Previous studies have reported lower rates of both non-diagnostic and false-negative cytology in ultrasound-guided fine needle aspiration biopsy [14, 15]. In our case, ultrasound-guided fine needle aspiration biopsy was performed twice (first biopsy: undeterminable and second biopsy: false-positive), which may depend partially on the low cellularity of myxoid liposarcoma.

We performed hemithyroidectomy for the single thyroid metastasis after resection of the primary myxoid liposarcoma. A previous report mentioned that thyroidectomy should be considered for thyroid metastasis when curability of the primary disease is expected and/or to improve quality of life by securing respiratory and swallowing function [1]. In our case, the primary tumor had already been resected and disease-free status was obtained by thyroidectomy which could improve survival in metastatic non-small round cell soft tissue sarcoma [16].

In our case, postoperative chemotherapy was not done because patient consent was not obtained. A meta-analysis showed that doxorubicin-based chemotherapy prolonged distant and overall recurrence in adults with localized, resectable soft tissue sarcoma, and the overall survival advantage was marginally determined [17]. However, in a randomized controlled trial, adjuvant chemotherapy with doxorubicin and ifosfamide showed no benefit in relapse-free survival or overall survival in patients with resectable soft tissue sarcoma [18]. Neoadjuvant and/or adjuvant chemotherapy may be one of the treatment options for patients with resectable primary soft tissue sarcoma, but the evidence is still controversial. Recent study showed that adjuvant treatment with anthracycline-based chemotherapy had survival benefits for high-risk patients with resectable primary tumor [19]. Doxorubicin-based chemotherapy has been also considered as a standard treatment for chemotherapy naïve advanced or metastatic soft tissue sarcoma patients [20, 21]. There is no strong evidence of the value of neoadjuvant and/or adjuvant chemotherapy for patients with resectable metastasis of soft tissue sarcoma, but doxorubicin-based chemotherapy was a treatment option for our patient. The overall response rates were reported as 43.2% using Response Evaluation Criteria in Solid Tumors (RECIST) and 86.5% using Choi criteria after treatment of first-line doxorubicin and ifosfamide in patient with myxoid liposarcoma [22].

There were some reports about the periods to recurrence in myxoid/round cell liposarcoma. Moreau et al. reported that the 5- and 10-year metastasis-free survival were 84 and 77% for myxoid liposarcoma and 69 and 46% for round cell liposarcoma, respectively [3]. In our sarcoma group, disease-free survival was 77% at both 5 and 10 years in pure myxoid liposarcoma [11]. Although postoperative recurrence after 5 years was rare, it would be reasonable to observe the follow-up for 10 years after surgery in patients with myxoid liposarcoma.

Finally, thyroid metastasis of soft tissue sarcoma is a very rare condition, but it should be considered especially in liposarcoma. Thyroidectomy may be optimal for the treatment of a single metastasis in the thyroid grand.

Summing up, even though thyroid metastasis of soft tissue sarcoma is very rare, we should consider the possibility of thyroid gland metastasis in patients with soft tissue sarcoma in whom a growing mass in the thyroid gland is observed.

## Abbreviations

CT: Computed tomography
MRI: Magnetic resonance imaging
RECIST: Response Evaluation Criteria in Solid Tumors
